# Spatio-temporal analysis of the main dengue vector populations in Singapore

**DOI:** 10.1186/s13071-020-04554-9

**Published:** 2021-01-11

**Authors:** Haoyang Sun, Borame L Dickens, Daniel Richards, Janet Ong, Jayanthi Rajarethinam, Muhammad E. E. Hassim, Jue Tao Lim, L. Roman Carrasco, Joel Aik, Grace Yap, Alex R. Cook, Lee Ching Ng

**Affiliations:** 1 Saw Swee Hock School of Public Health, National University of Singapore and National University Health System, 12 Science Drive 2, Singapore, 117549 Republic of Singapore; 2 Natural Capital Singapore, Singapore-ETH Centre, ETH Zurich, Singapore, Singapore; 3 Environmental Health Institute, National Environment Agency, Singapore, Singapore; 4 Centre for Climate Research Singapore, Meteorological Service Singapore, National Environment Agency, Singapore, Singapore; 5 Department of Biological Sciences, National University of Singapore, Singapore, Singapore; 6 School of Biological Sciences, Nanyang Technological University, Singapore, Singapore

**Keywords:** *Aedes*, Dengue, Vector control, Spatio-temporal modeling

## Abstract

**Background:**

Despite the licensure of the world’s first dengue vaccine and the current development of additional vaccine candidates, successful *Aedes* control remains critical to the reduction of dengue virus transmission. To date, there is still limited literature that attempts to explain the spatio-temporal population dynamics of *Aedes* mosquitoes within a single city, which hinders the development of more effective citywide vector control strategies. Narrowing this knowledge gap requires consistent and longitudinal measurement of *Aedes* abundance across the city as well as examination of relationships between variables on a much finer scale.

**Methods:**

We utilized a high-resolution longitudinal dataset generated from Singapore’s islandwide Gravitrap surveillance system over a 2-year period and built a Bayesian hierarchical model to explain the spatio-temporal dynamics of *Aedes aegypti* and *Aedes albopictus* in relation to a wide range of environmental and anthropogenic variables. We also created a baseline during our model assessment to serve as a benchmark to be compared with the model’s out-of-sample prediction/forecast accuracy as measured by the mean absolute error.

**Results:**

For both *Aedes* species, building age and nearby managed vegetation cover were found to have a significant positive association with the mean mosquito abundance, with the former being the strongest predictor. We also observed substantial evidence of a nonlinear effect of weekly maximum temperature on the *Aedes* abundance. Our models generally yielded modest but statistically significant reductions in the out-of-sample prediction/forecast error relative to the baseline.

**Conclusions:**

Our findings suggest that public residential estates with older buildings and more nearby managed vegetation should be prioritized for vector control inspections and community advocacy to reduce the abundance of *Aedes* mosquitoes and the risk of dengue transmission.
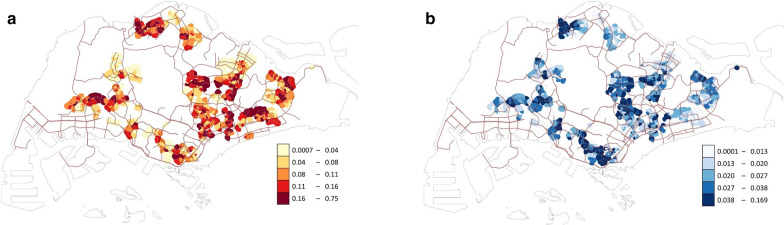

## Background

Dengue fever is a rapidly emerging vector-borne disease mainly transmitted by *Aedes aegypti* and *Aedes albopictus* [1], causing an estimated number of 390 million infections per year worldwide [2]. Clinical manifestations of dengue infection range from mild fever to potentially lethal complications such as dengue shock syndrome [1]. Despite the licensure of the world’s first dengue vaccine and the current development of additional vaccine candidates [3], successful vector control remains critical to the reduction of dengue virus transmission [4]. Moreover, the benefits of *Aedes* population control extend beyond dengue infection prevention alone, given the multiple diseases that can be transmitted by these mosquito species, such as Zika, chikungunya, and yellow fever.

Previous work has yielded important insights into the behaviors and ecology of the main dengue vectors. Both *Aedes* species can easily disperse throughout areas with ~ 300 m radius to seek oviposition sites [5]. The *Ae. aegypti* mosquitoes in particular have become a highly efficient vector for dengue transmission owing to their skip oviposition behavior (i.e. deposit eggs from the same batch in multiple sites), desiccation-resistant eggs, preference for human biting, multiple feeds per gonotrophic cycle, and adaptation to reside and breed in human habitats, among other factors [6]. The *Ae. albopictus* mosquitoes were found to have a relatively lower contribution to the reported dengue cases overall despite their high competence for dengue transmission, which is primarily attributed to aspects of their ecology [7]. Both environmental and anthropogenic factors can exert an important influence on the distribution of *Aedes* mosquitoes [8–12], and modeling studies have been carried out to map the suitability and distribution of the main dengue vectors at a global scale [10–12]. However, there is still very limited literature that attempts to explain the spatio-temporal population dynamics of *Aedes* mosquitoes within a single city [13], which hinders the development of more effective citywide vector control strategies. To bridge this knowledge gap requires consistent and longitudinal measurement of *Aedes* abundance across the city [13] as well as examination of relationships between variables at a much finer scale.

As an island city-state lying 1° north of the equator, Singapore faces regular dengue outbreaks with the four dengue virus serotypes co-circulating all year round [14]. The low herd immunity [15], coupled with the tropical climate and highly urbanized environment, poses challenges to the nation’s dengue control program [16]. As part of Singapore’s vector control program, the National Environment Agency has conducted regular inspections of homes and surrounding areas all year round to remove mosquito-breeding habitats and mobilized the community and stakeholders to minimize instances of stagnant water [17]. Vector control activities were also ramped up in dengue cluster areas, with space sprays used for adulticiding. To monitor the spatio-temporal trend of the adult *Aedes* abundance in Singapore, the National Environment Agency also established an islandwide Gravitrap surveillance system in 2017, with over 50,000 Gravitraps deployed in the public housing estates across the island [18, 19], which accommodate ~ 80% of the resident population [20]. The weekly mean catch per trap for each species provides an indication of the *Aedes* abundance around each specific residential location and each time point, which is presumed to be closely associated with an individual’s risk of exposure to mosquito bites inside or around homes and also much less susceptible to the measurement bias encountered in non-systematic breeding sites inspection [21]. To facilitate resource planning for Singapore’s vector control, we used the longitudinal dataset generated from the islandwide Gravitrap surveillance system during 2017–2018 as well as a wide range of environmental and anthropogenic variables acquired from various sources to (1) explain the spatio-temporal dynamics of the *Ae. aegypti* and *Ae. albopictus* population in Singapore’s high-rise residential zones and (2) assess our model’s ability to predict *Aedes* abundance across space and generate forecasts up to 3 weeks ahead.

## Methods

### Data

*Aedes* mosquito data were collected fortnightly for each of the 552 sites from 2017–2018 [19], with odd-numbered blocks inspected 1 week and even-numbered blocks the next. Fortnightly collections were then halved to obtain the weekly numbers of *Ae. aegypti* and *Ae. albopictus* caught at each site respectively, which contained roughly equal numbers of odd- and even-numbered blocks. We created a 300-m buffer around each block based on Liew et al. [5], and for each site, all buffers were merged into a single polygon to be used for deriving zonal statistics for the environmental and anthropogenic variables.

The Singapore land classification map was generated at a resolution of 10 m using seven separate images from the Sentinel-2 satellite of the European Space Agency [22]. The collected images were taken on different dates to ensure the existence of cloud-free pixels for the whole of mainland Singapore based on a cloud cover classification algorithm [22]. With 309 labeled data points obtained manually using Google Earth, a random forest algorithm was used to produce seven land cover maps, excluding cloudy areas for each of the collected images [22]. The final classified land cover for each pixel was set to be the majority vote out of all the predictions, with an out-of-bag classification accuracy of 81% [22]. For each site, we derived the percentage of the buffer area covered by water, grass, forest, and managed vegetation (i.e. trees and shrubs with structure dominated by human management), respectively, setting “urban” as the reference level.

Data on waterbodies were extracted from OpenStreetMap [23]. We measured the distance to the nearest waterway from each block using ArcMap 10.6, which was then averaged within each site (similarly to the distance to the nearest water area). We also obtained Singapore’s drain line map from the Public Utilities Board. The total drain line density for each site was defined to be the total length of the drain lines falling within the corresponding buffer divided by the buffer area. In addition, the average age of buildings for each site was computed using lease commencement year data collected from the Singapore Land Authority [24].

Weekly mean, maximum, and minimum temperature and mean relative humidity were obtained from a total of 21 weather stations installed by the National Environment Agency. For each climatic variable and each week, we fitted a thin plate spline surface to produce an interpolated value for each site. Weekly raster maps of total precipitation were obtained from the Meteorological Service Singapore at ~ 500 m × 500 m resolution, and for each site and each week, all the pixel values within the corresponding buffer were averaged. All the aforementioned explanatory variables were standardized to zero mean and unit variance, and a quadratic term for each of the standardized temperature variables was created to examine nonlinear effects [25].

### Statistical analyses

To understand the direction and strength of associations between *Aedes* abundance and different environmental and anthropogenic variables, we first computed the pairwise Pearson correlation coefficients for the full set of covariates and removed redundant variables using a threshold of ± 0.6 to avoid collinearity. A Bayesian spatio-temporal model was created, where we assumed that the number of *Ae. aegypti* or *Ae. albopictus* caught at site $i$ during week $t$ ($y_{it}$) followed a negative binomial distribution with mean $\mu_{it}$ and dispersion parameter $r$, namely:

$$y_{it} \sim NB\left( {\mu_{it} , r} \right),$$

where

$$\log \left( {\mu_{it} } \right) = \log \left( {E_{it} } \right) + b_{0} + \mathop \sum \limits_{j} \beta_{j} x_{ij} + \mathop \sum \limits_{k} \gamma_{k} w_{itk} + u_{i} + v_{{PA_{i} }} + \phi_{t} .$$

In the equation above, $E_{it}$ denotes the number of Gravitraps present at site $i$ during week $t$, $b_{0}$ the intercept, $x_{ij}$ the spatial variables of site $i$, and $w_{itk}$ all the weekly weather measurements of site $i$ between 1 and 3 weeks prior to week $t$. We included an unstructured spatial effect $u_{i}$ for each site and an extra term $v_{{PA_{i} }}$ for the corresponding planning area (each containing 20 sites on average, with an interquartile range of 8–26) to account for additional spatial dependence. The temporally structured effect $\phi_{t}$ was assumed to follow a random walk with a maximum order of two. Throughout this article, we used $t = 1$ to denote the first epidemiological week of 2017 and $t = 104$ the last epidemiological week of 2018. All the model parameters were assigned a minimally informative prior (refer to Table 2 caption), and parameter estimation was performed using Integrated Nested Laplace Approximation [26, 27], with 95% credible intervals (CrI) computed to summarize the uncertainty in each model parameter.

For each species, both the optimal order of the random walk for the temporally structured effect and the time lag of the weather variables were selected based on the deviance information criterion. Further variable subset selection was not implemented at this stage to avoid biased parameter estimates resulting from sequential comparisons, since the primary aim of our Bayesian spatio-temporal model was to infer the associations between *Aedes* abundance and all the different environmental and anthropogenic variables considered in this study.

Next, we used cross-validation to assess how accurately one can predict *Aedes* abundance across space. Here we treated the total number of *Ae. aegypti* or *Ae. albopictus* caught at each site during 2017–2018 as the response variable, with the log transformation of the total number of trap-week units as an offset. Only spatial fixed effects and the planning area random effect were included in the model, and a backwards elimination procedure was implemented for fixed effects variable selection using the Akaike information criterion during model training. Two forms of cross-validations were performed, namely, leave-one-site-out and leave-one-planning-area-out, where in each case a baseline prediction of the mean catch per trap per week was generated for each site, to be used as a benchmark to assess whether the model can indeed yield a higher prediction accuracy. In the former case, each time a site $i$ was left out for testing, its baseline prediction was defined as the observed mean catch per trap per week of the site that was geographically the closest to site $i$. In the latter case, the baseline prediction for each site $i$ was defined as the observed mean catch per trap per week averaged across all the sites within the planning area that was geographically the closest to site $i$ but did not contain site $i$.

Finally, we evaluated the contribution of weather variables to the improvement of the weekly *Aedes* abundance forecast accuracy up to 3 weeks ahead. Using $t_{C}$ to denote the current time point, we treated the mean catch per trap of each site at week $\left( {t_{C} + \Delta t} \right) \left( {\Delta t = 1,2, {\text{or }} 3} \right)$ as the response variable, and each was regressed upon all the weather and/or entomological (i.e. *Aedes* abundance) covariates at weeks $t_{C}$, $\left( {t_{C} - 1} \right)$ and $\left( {t_{C} - 2} \right)$. Here, the entomological covariates were always included as autoregressive terms, with the additional inclusion of weather covariates in an alternative model to assess the resulting change in the out-of-sample forecast errors. Due to the large number of lagged weather variables included in the alternative model, we used the least absolute shrinkage and selection operator to perform variable selection during model training. For each site and each species, we derived the out-of-sample *Aedes* abundance forecast accuracy at week $\left( {t^{*} + \Delta t} \right)$ for all combinations of $t^{*} \in \left\{ {55, 60, \ldots , 95, 100} \right\}$ and $\Delta t \in \left\{ {1,2,3} \right\}$, respectively, with the model being trained on all the historical data points of that site with $t_{C} < t^{*}$. The corresponding baseline forecast was defined as the observed *Aedes* abundance of that site at week $t^{*}$, and hence we will also refer to $t^{*}$ as the baseline time point.

## Results

**Fig. 1:**
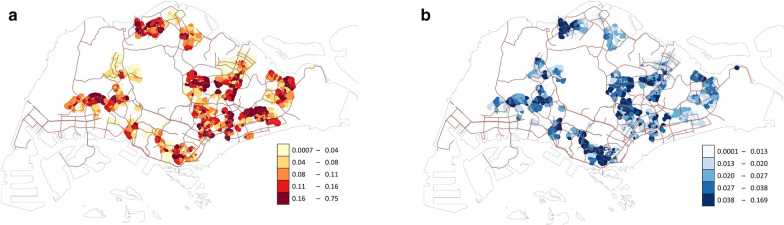
Observed *Aedes* abundance (mean catch per trap per week) of each site during 2017–2018: (**a**) *Ae. aegypti* and (**b**) *Ae. albopictus*. We first created a 300 m buffer around each block, and all buffers for each site were merged into a single polygon, which was then colored according to the observed *Aedes* abundance value

In total, 4,923,456 trap-week units of observation were obtained, with 495,638 *Ae. aegypti* and 132,533 *Ae. albopictus* caught during the entire study period. For both species, we observed a marked difference in the *Aedes* abundance across space, with the mean catch per trap per week at some sites exceeding fivefold that at some other sites (Fig. 1). For example, only an average of 0.05 *Ae. aegypti* mosquitoes were caught per trap per week at a site within the Clementi area during the study period, in contrast to 0.28 at a site in Tampines. Operationally, these data are updated on a weekly basis to provide policy makers with an indication of which areas may require more vector control to mitigate the risk of dengue transmission. The mean and standard deviation of the site-level mean catch per trap per week were 0.102 and 0.074 for *Ae. aegypti* and 0.027 and 0.019 for *Ae. albopictus* (Table 1 contains the summary statistics of all the variables in this study, and visualizations of selected variables across space or time can be found in Additional file 1: Supporting information).

**Table 1:** Summary statistics of all the variables included in the study

Variables	Mean (SD)
Mean number of mosquitoes caught per trap per week (*Ae. aegypti*)	0.102 (0.074)
Mean number of mosquitoes caught per trap per week (*Ae. albopictus*)	0.027 (0.019)
Forest cover (% of 300 m radius buffer)	5.6 (5.2)
Water cover (% of 300 m radius buffer)	2.0 (3.2)
Grass cover (% of 300 m radius buffer)	8.6 (4.8)
Managed vegetation cover (% of 300 m radius buffer)	25.4 (7.8)
Drain line density (km^−1^)	9.7 (6.9)
Distance to waterway (m)	685 (490)
Distance to water area (m)	613 (390)
Building age (years as of 2018)	28 (10)
Weekly mean temperature (°C)	27.9 (0.9)
Weekly maximum temperature (°C)	33.1 (0.9)
Weekly minimum temperature (°C)	23.9 (1.0)
Weekly mean relative humidity (%)	80.5 (4.1)
Weekly total precipitation (mm)	51.6 (63)

**Fig. 2:**
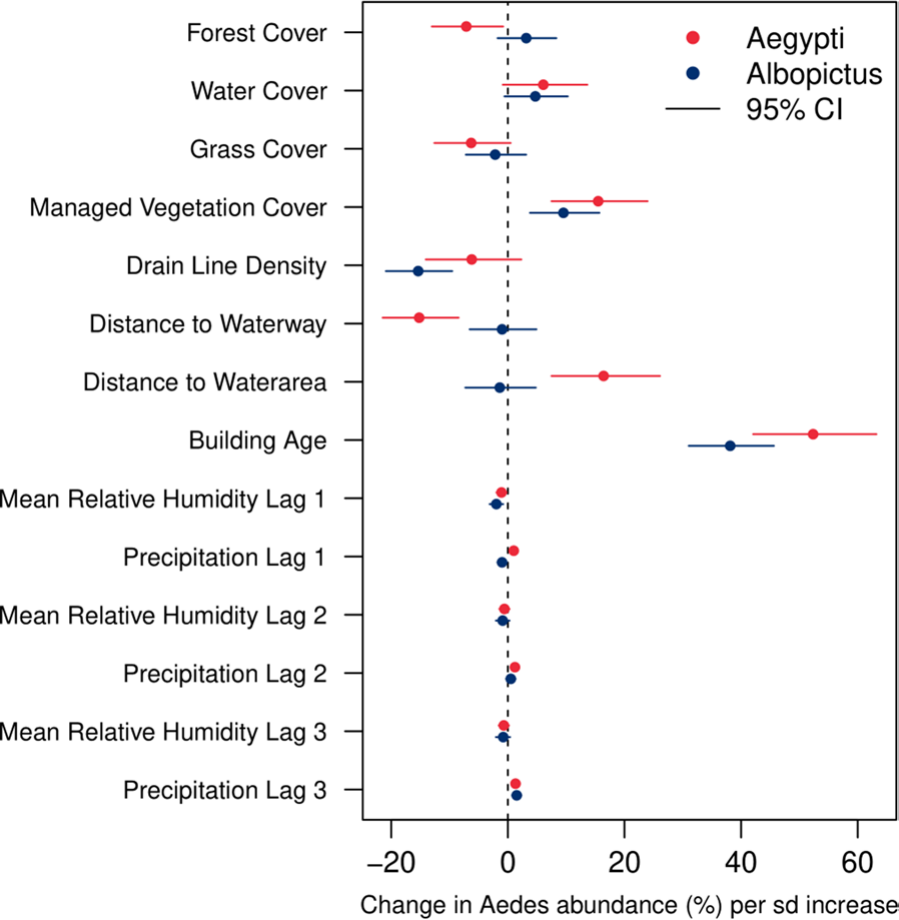
Estimated percentage change in the expected value of *Aedes* abundance (weekly mean catch per trap) due to a 1-SD increase in each covariate when all the other variables were held constant. Filled circles denote the posterior median estimates, and the solid lines denote the 95% credible intervals. The standard deviation of each covariate can be found in Table 1, and the estimated effects of lagged temperature covariates on the predicted *Aedes* abundance were visualized in Fig. 3

Based on the spatio-temporal model estimates, both nearby managed vegetation cover and building age were found to have a direct association with the abundance of both species (Table 2 and Fig. 2). On average, we estimated that a 1-SD (10 years) increase in the average age of buildings was associated with a 52.3% (95% CrI: 42.0%–63.2%) increase in the *Ae. aegypti* abundance and a 38.1% (95% CrI: 31.0%–45.6%) increase in the *Ae. albopictus* abundance at the site level, when all the other variables were held constant (Fig. 2). For forest cover and distance to water area, the signs of the *point* estimates were found to be opposite between the two mosquito species, although the 95% credible interval may contain the null effect in some cases (Table 2 and Fig. 2). Even after controlling for all the fixed effects, substantial heterogeneity of *Aedes* abundance remained both between sites and between planning areas, as shown by the standard deviation estimates of the spatial random effects (Table 2).

**Table 2:** Posterior estimates of the Bayesian spatio-temporal model parameters^§^

		*Ae. aegypti*			*Ae. albopictus*	
	2.5th percentile	50th percentile	97.5th percentile	2.5th percentile	50th percentile	97.5th percentile
Forest cover (within 300 m radius buffer)	− 0.140	− 0.074	− 0.008	− 0.018	0.031	0.080
Water cover (within 300 m radius buffer)	− 0.009	0.059	0.128	− 0.006	0.046	0.098
Grass cover (within 300 m radius buffer)	− 0.135	− 0.065	0.005	− 0.075	− 0.022	0.031
Managed vegetation cover (within 300 m radius buffer)	0.072	0.144	0.215	0.037	0.091	0.146
Drain line density (within 300 m radius buffer)	− 0.152	− 0.064	0.023	− 0.235	− 0.167	− 0.100
Distance to waterway	− 0.242	− 0.165	− 0.088	− 0.068	− 0.010	0.048
Distance to water area	0.072	0.152	0.232	− 0.076	− 0.014	0.047
Building age	0.351	0.421	0.490	0.270	0.323	0.376
Max temperature lag 1	− 0.037	− 0.027	− 0.018	− 0.027	− 0.016	− 0.004
(Max temperature lag 1)^2^	− 0.008	− 0.004	0.000	− 0.010	− 0.005	− 0.001
Mean relative humidity lag 1	− 0.020	− 0.011	− 0.002	− 0.032	− 0.020	− 0.008
Precipitation lag 1	0.003	0.010	0.016	− 0.018	− 0.010	− 0.002
Max temperature lag 2	− 0.040	− 0.031	− 0.021	− 0.029	− 0.017	− 0.005
(Max temperature lag 2)^2^	− 0.008	− 0.005	− 0.001	− 0.010	− 0.005	0.000
Mean relative humidity lag 2	− 0.015	− 0.006	0.003	− 0.021	− 0.009	0.003
Precipitation lag 2	0.006	0.012	0.018	− 0.003	0.005	0.013
Max temperature lag 3	− 0.032	− 0.023	− 0.013	− 0.025	− 0.013	− 0.002
(Max temperature lag 3)^2^	− 0.009	− 0.006	− 0.002	− 0.010	− 0.005	0.000
Mean relative humidity lag 3	− 0.016	− 0.007	0.002	− 0.021	− 0.008	0.004
Precipitation lag 3	0.007	0.013	0.020	0.007	0.015	0.023
Unstructured spatial effect (site)^*^	0.603	0.642	0.686	0.443	0.475	0.508
Unstructured spatial effect (planning area)^*^	0.383	0.514	0.708	0.240	0.337	0.487
Temporally structured effect^**^	0.048	0.058	0.071	0.076	0.092	0.113
Negative binomial size parameter	6.681	6.863	7.049	12.877	13.679	14.597

All the environmental and anthropogenic variables were standardized to zero mean and unit variance prior to model fitting, and a quadratic term for each of the standardized temperature variables was also created to introduce nonlinear effects. Estimates are posterior median and equal tailed 95% credible intervals

^*^Refers to the posterior estimate of the standard deviation of the spatial random effect

^**^ Refers to the posterior estimate of the standard deviation of the independent second-order increment in the temporally structured effect

^§^Each regression parameter (i.e. intercept and the coefficients of the fixed effects) was assigned a normal prior ${\text{N}}\left( {0,5^{2} } \right)$. We assumed a ${\text{logGamma}}\left( {1, 0.01} \right)$ prior on the logarithm of the precision of the spatial random effects and independent second-order increment in the temporally structured effect. The default penalized complexity prior in R-INLA was specified for the logarithm of the negative binomial size parameter

**Fig. 3:**
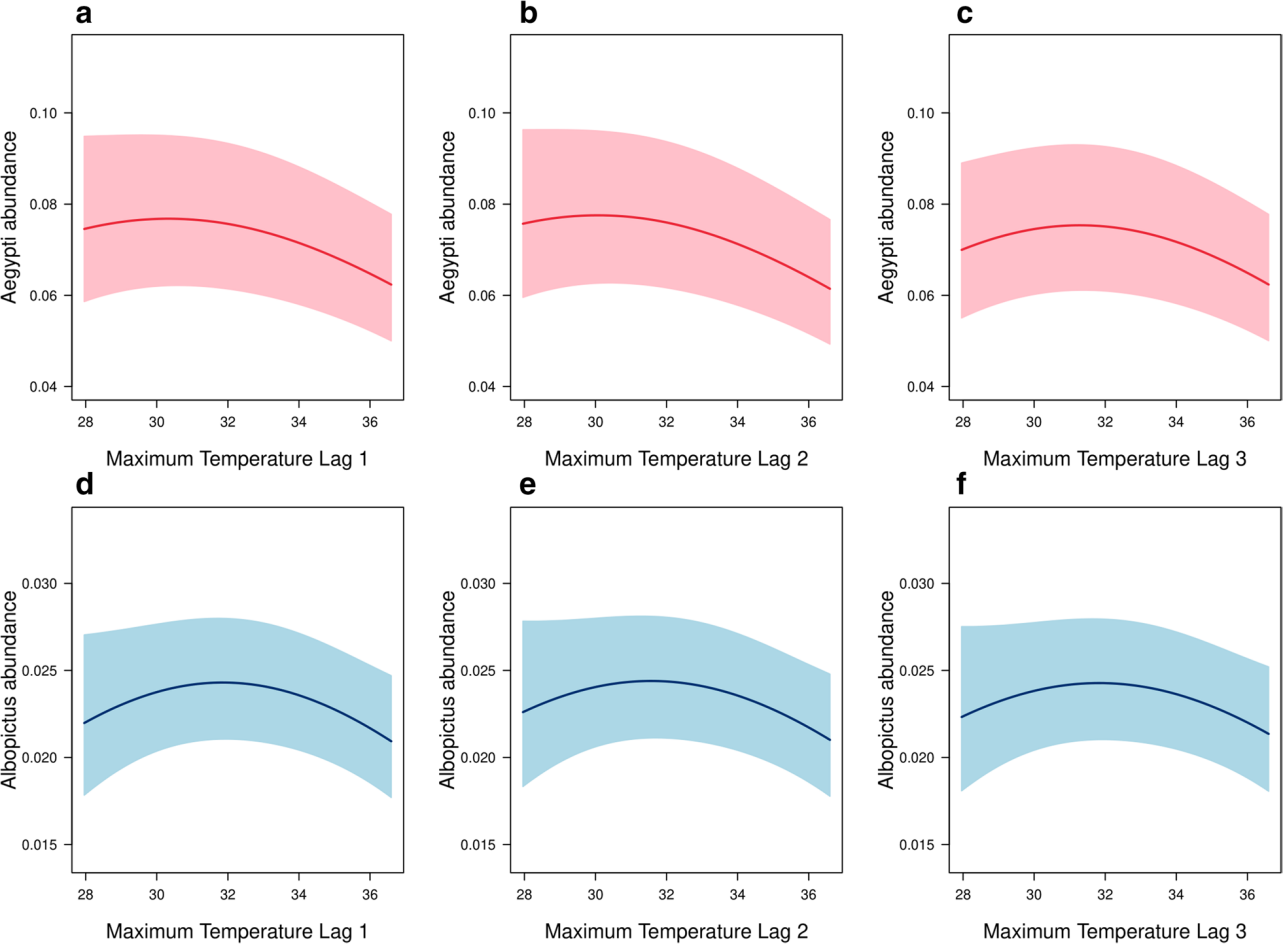
Predicted values of *Aedes* abundance (weekly mean catch per trap) at different values of lagged weekly maximum temperature with all the other variables held fixed at their average values. Solid lines denote the posterior median estimates and the shaded areas denote the 95% prediction bands

For both species, inclusion of weather measurements in all the past 3 weeks together with a random walk model of order 2 for the temporally structured effect yielded the lowest deviance information criterion. However, compared with the spatial covariates, the weather covariates were estimated to have a relatively limited impact on the variation of adult *Aedes* abundance in the context of Singapore (Table 2 and Fig. 2). The 95% credible intervals of the quadratic term coefficients for the weekly maximum temperature were away from zero for both species and all time lags (Table 2), and the *Aedes* abundance was estimated to first increase and then decrease as we varied the lagged weekly maximum temperature from 28.0 °C to 36.6 °C while holding the other variables constant (Fig. 3). Specifically, with all other covariates held constant, the median estimate of *Ae. aegypti* abundance peaked at 30.3 °C, 30.0 °C, and 31.3 °C for weekly maximum temperature measured at 1, 2, and 3 weeks’ lag, respectively. Similarly, the turning points were 31.9 °C, 31.6 °C, and 31.9 °C for *Ae. albopictus*. Out of all the covariates collected in this study, only weekly mean and minimum temperatures were removed from the Bayesian spatio-temporal model during collinearity assessment.

**Fig. 4:**
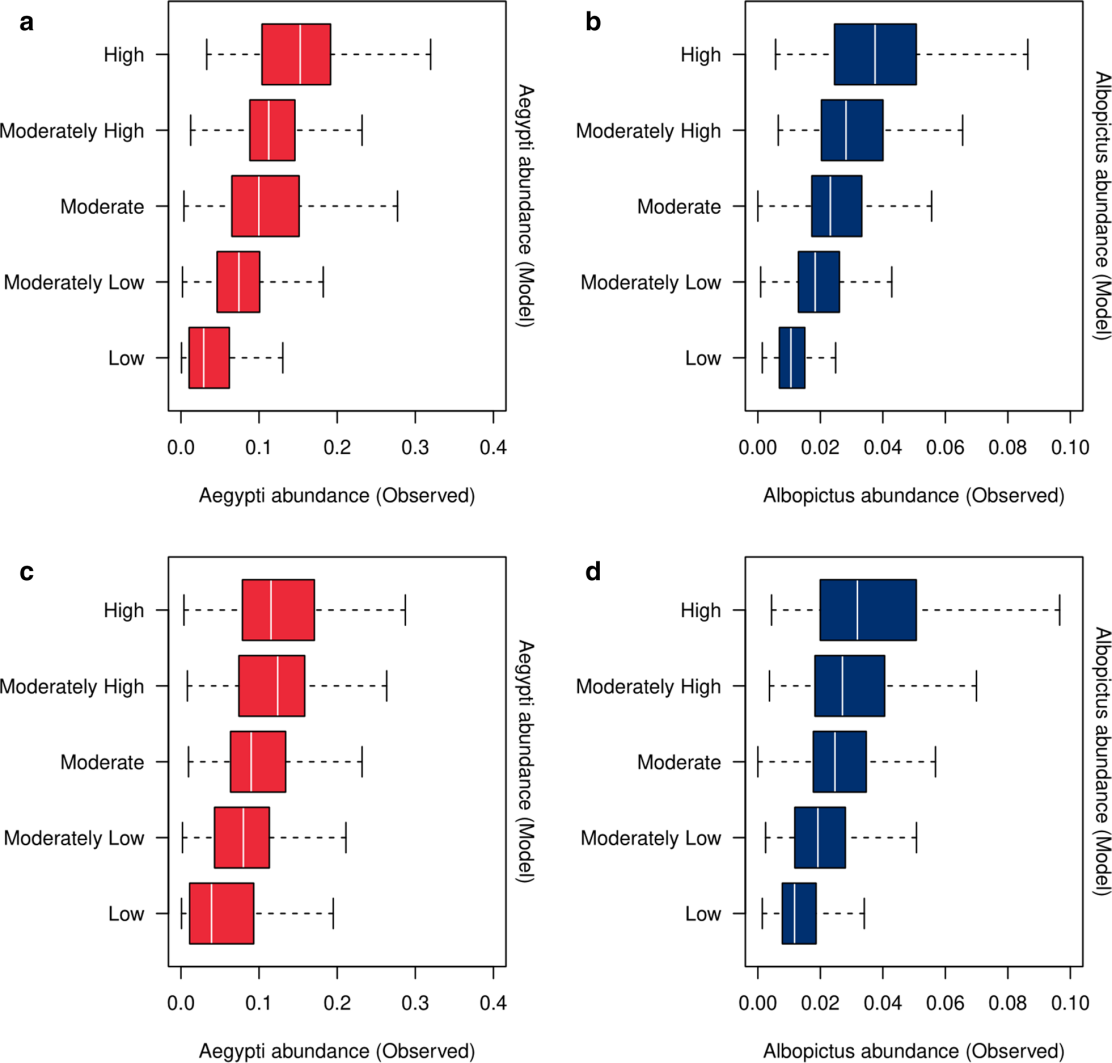
Box-plots of the observed site-level *Aedes* abundance (mean catch per trap per week) during 2017–2018 within each quintile based on the out-of-sample model predictions: (**a**), (**b**) leave-one-site-out cross validation and (**c**), (**d**) leave-one-planning-area-out cross validation. A small number of extreme values were omitted from the graph for clarity

In both leave-one-site-out and leave-one-planning-area-out cross validations, which were performed to assess predictive accuracy across space, there was an overall increasing trend in the observed site-level *Aedes* abundance as we moved from the lowest to the highest quintile based on the out-of-sample model predictions (Fig. 4). Except for the leave-one-site-out cross validation of the model for *Ae. aegypti*, there was a modest and statistically significant reduction in the mean absolute prediction error of the model compared with the baseline prediction (Table 3). Likewise, a modest and statistically significant reduction in the out-of-sample forecast error was observed for models forecasting 2- or 3-week ahead *Aedes* abundance, regardless of species or whether we included weather covariates as additional predictors (Table 4). Our model, however, did not outperform the 1-week ahead baseline forecast (Table 4), owing to the fortnightly mosquito collection and the subsequent conversion to weekly data (details described in Methods), which caused data points at adjacent weeks to share 50% of the information in common. Notably, we found that in all cases the additional inclusion of lagged weather covariates did not improve the out-of-sample forecast accuracy compared with a simple model that only included autoregressive terms as predictors (Table 4).

**Table 3:** Percentage reduction in the out-of-sample mean absolute prediction error of the model compared with the baseline prediction

	Leave-one-site-out	Leave-one-planning-area-out
*Ae. aegypti*	3.6% (*p* = 0.477)	9.3% (*p* = 0.006)
*Ae. albopictus*	17.5% (*p *< 0.001)	14.1% (*p* < 0.001)

Under both types of spatial cross validation, the mean absolute prediction error was derived by averaging the absolute prediction errors across all sites. The *p*-values were obtained based on a two-sided paired *t* test

**Table 4:** Percentage reduction in the out-of-sample mean absolute forecast error of the model compared with the baseline forecast

		1 week ahead	2 weeks ahead	3 weeks ahead
Weather covariates included	*Ae. aegypti*	− 10.0% (*p *< 0.001)	6.5% (*p *< 0.001)	10.7% (*p *< 0.001)
	*Ae. albopictus*	− 18.5% (*p *< 0.001)	7.8% (*p *< 0.001)	10.3% (*p *< 0.001)
Weather covariates not included	*Ae. aegypti*	0.3% (*p* = 0.764)	8.3% (*p *< 0.001)	10.8% (*p *< 0.001)
	*Ae. albopictus*	− 4.9% (*p *< 0.001)	9.8% (*p *< 0.001)	11.0% (*p *< 0.001)

The mean absolute forecast error was derived by averaging the absolute forecast errors across all sites and baseline time points. The *p*-values were obtained based on a two-sided paired t-test

## Discussion

This study examined the spatial and temporal variation of the main dengue vectors in Singapore’s high-rise public residential zones in relation to a wide range of environmental and anthropogenic variables. The insights derived from this study further add to previous work that aimed to understand *Aedes* ecology in the local context and can facilitate the formulation of more effective vector control strategies in the future. Our model performance also suggests the potential use of spatio-temporal mapping as a tool to improve the understanding of the *Aedes* distribution in other cities or countries, where intensive entomological surveillance may be harder to achieve.

We found that the majority of our spatial covariates had an at least borderline significant association with the *Aedes* abundance (i.e. the 95% credible intervals did not/barely overlap zero). In particular, building age was shown to be the strongest predictor. This might be due to a combination of factors, including infrastructural degradation and the water storing practices associated with the sociodemographic profile of residents that result in more instances of water stagnation that can breed mosquitoes. For both species, we estimated that an increase in the managed vegetation cover within the buffer area was associated with a substantial rise in the mean vector abundance, likely owing to the increased availability of water in leaf axils, leaf litter, and discarded receptacles hidden in foliage and tree holes, which supports mosquito breeding. Unlike managed vegetation cover, both forest and grass covers were found to be negatively associated with the abundance of *Ae. aegypti*. This was not unexpected given that *Ae. aegypti* prefers highly urbanized areas where it can breed in artificial containers. On the other hand, there was a positive association between forest cover and *Ae. albopictus* abundance based on the point estimate, which is consistent with the existing knowledge of the vector’s ecology [28].

Our analysis showed a borderline significant negative association between drain line density and the abundance of *Ae. aegypti* in contrast to the positive correlation reported by Seidahmed et al. [29]. This discrepancy can be due to a number of reasons: for example, the study conducted by Seidahmed et al. was restricted to a small area of Singapore, and results may be confounded by the different housing types with different demography [29], whereas this study used 2-year data collected from the same type of housing areas across the island. Moreover, the number of *Aedes* mosquitoes caught inside the high-rise residential buildings and the number of outdoor breeding habitats can be impacted by drain line density in different ways. Since perimeter drains are known to be the most common breeding habitats of *Aedes* in Singapore’s public areas according to routine inspections [30], their abundance could simultaneously decrease the per-mosquito probability of looking for oviposition sites inside residential buildings and increase the total number of *Aedes* mosquitoes in the nearby public area. Thus, our estimate is likely to be a reflection of the resulting net effect and similarly for the estimated effects of other spatial covariates such as nearby managed vegetation cover.

Previous work has highlighted the challenges of mapping the spatial distribution of *Aedes* mosquitoes for operational dengue vector control [31]. In particular, predictors that can be informative across an entire continent or a sufficiently large country may lose predictive power within the confines of a single city [31]. While we did observe substantial evidence of a non-zero association between many spatial covariates and *Aedes* abundance in our analysis, the estimated standard deviations of the spatial random effects remained large, suggesting substantial unexplained heterogeneity across space. Hence, entomological surveillance remains critical to generating knowledge of *Aedes* abundance in the field to inform vector control. On the other hand, we found that in most cases the model performed significantly better than the baseline at predicting mosquito abundance at new locations, based on the spatial variables at those locations, suggesting that statistical modeling can still serve as a complementary tool to refine our understanding of the *Aedes* abundance at locations where entomological data are unavailable and hence to identify additional locations that may require enhanced vector control. It should be noted that the model’s improvement in prediction accuracy over the baseline was found to be smaller for *Ae. aegypti* than *Ae. albopictus*, despite the former being the most important dengue vector in Singapore. This finding may be explained by the ecology of *Ae. aegypti*, which is a container breeder that is subject to the vagaries of human behavior; adherence to household practices to prevent breeding is hard to measure and may vary spatially, rendering spatial modeling much more challenging.

There is abundant literature on how different weather variables regulate the population dynamics of *Aedes* via influencing mosquito habitat availability, development, survival, and reproduction [9]. In this study, however, we estimated the effects of all the lagged weather variables on the observed *Aedes* abundance to be very minimal, which can be owing to the restricted range of the weather variables in the context of Singapore as well as vector control activities that were typically ramped up during higher breeding seasons. The assessment of the out-of-sample forecast errors, too, shows that the additional inclusion of weather covariates did not improve the accuracy of the *Aedes* abundance forecasts, and a simple model with autoregressive terms alone could yield a modest and statistically significant improvement in the 2- and 3-week-ahead forecasts over the baseline. While these results may suggest that weather had a negligible impact on *Aedes* abundance in Singapore, it should be noted that this study was conducted in public housing estates and detected mosquitoes that may have hatched nearby, and the relationship between weather and outdoor breeding habitats may be more complex than was identified herein. A longitudinal entomological study in the Geylang neighborhood of Singapore found that the outdoor *Aedes* population was likely to be shaped by rainfall through a monsoon-driven sequence of flushing, drying and return of breeding habitats [32]. Taken together, these results suggest the differential impacts of weather on the *Aedes* population dynamics and hence the potential risk of exposure to mosquito bites in different settings, i.e. *Aedes* abundance inside and nearby public housing estates may be less sensitive to changes in weather compared with outdoor abundance.

Our results need to be interpreted in the light of the following limitations. First, we were unable to account for the effects of vector control programs on the observed *Aedes* abundance across space and time. Regulatory inspections and community efforts aimed at removing larval habitats, as well as chemical control to reduce adult mosquito populations, usually peak during higher vector breeding/dengue transmission seasons, and this could to some extent mask the true association between different weather variables and the *Aedes* abundance, with the confounding bias difficult to quantify or adjust for. Besides, there could be a residual spatial dependence structure in our data due to factors such as potential ongoing expansion of *Aedes* mosquitoes, which may have been coincidentally absorbed by certain spatial covariates because of confounding. Nonetheless, this issue was assessed via a leave-one-planning-area-out cross-validation framework with baseline predictions created to serve as a benchmark for model performance comparison, and results suggest that the spatial covariates could indeed enhance the out-of-sample predictive accuracy. In addition, our parameter estimates were derived based on mosquito data collected from Singapore’s high-rise public residential zones and thus should not be used for extrapolation to low-rise houses. Previous work has suggested that the risks of indoor breeding of *Aedes* mosquitoes could be highly dependent on the accommodation type in Singapore [29]. In early 2020, the National Environment Agency extended the deployment of Gravitraps to private landed residential estates [33], and as more data are being generated, this will shed further light on how *Aedes* abundance differs between high- and low-rise residential zones across the island.

## Conclusions

Our study has demonstrated the potential and challenges of spatio-temporal modeling for improving the understanding of the main dengue vectors’ ecology and provided empirical evidence to guide the refinement of vector control strategies in the context of Singapore. Our findings suggest that public residential estates with older buildings and more nearby managed vegetation should be prioritized for vector control inspections and community advocacy to reduce the abundance of *Aedes* mosquitoes and the risk of dengue transmission. The insights obtained from this study could also be helpful to inspire future studies that attempt to understand the spatio-temporal dynamics of the dengue vector population at a city scale, particularly in settings where entomological surveillance data are less abundant and thus require modeling to further narrow the knowledge gap.

## Supplementary information

**Additional file 1: Figures.**

## Data Availability

The datasets used and analyzed during the current study are available from the corresponding authors upon reasonable request and with the permission of the National Environment Agency or Singapore-ETH Centre.
