# Supplementary material for: Spatio-temporal analysis of the main dengue vector populations in Singapore
Source: Parasit Vectors. 2021 Jan 11;14:41. doi: 10.1186/s13071-020-04554-9 (PMC7802191; doi:10.1186/s13071-020-04554-9)

Spatio-temporal analysis of the main dengue vector populations in Singapore

Haoyang Sun^1, §^, Borame Dickens^1^, Daniel Richards^2^, Janet Ong^3^, Jayanthi Rajarethinam^3^, Muhammad E. E. Hassim^4^, Jue Tao Lim^1^, L Roman Carrasco^5^, Joel Aik^3^, Grace Yap^3^, Alex R Cook^1, §^, Lee Ching Ng^3, 6^

^1^ Saw Swee Hock School of Public Health, National University of Singapore and National University Health System, Singapore

^2^ Natural Capital Singapore, Singapore-ETH Centre, ETH Zurich, Singapore

^3^ Environmental Health Institute, National Environment Agency, Singapore, Singapore

^4^ Centre for Climate Research Singapore, Meteorological Service Singapore, National Environment Agency, Singapore, Singapore

^5^ Department of Biological Sciences, National University of Singapore, Singapore

^6^ School of Biological Sciences, Nanyang Technological University, Singapore, Singapore

^§^ Correspondence to:

Haoyang Sun. Saw Swee Hock School of Public Health, National University of Singapore, 12 Science Drive 2, Singapore 117549, Republic of Singapore. Email: ephsunh@nus.edu.sg.

Alex R Cook. Saw Swee Hock School of Public Health, National University of Singapore, 12 Science Drive 2, Singapore 117549, Republic of Singapore. Email: ephcar@nus.edu.sg.

Supporting Information

**Figure S1: Weekly mean catch per trap for *Aedes aegypti* (red) and *Ae. albopictus* (blue) at Bukit Batok Street 52, Jurong West Avenue 1, Tampines Street 21, and Yishun Avenue 9.** Week 1 refers to the first epidemiological week of 2017, and week 104 the last epidemiological week of 2018. Due to the large number of sites (n=552) in our study, we picked these four sites to visualize *Aedes* abundance over time for demonstration purposes.


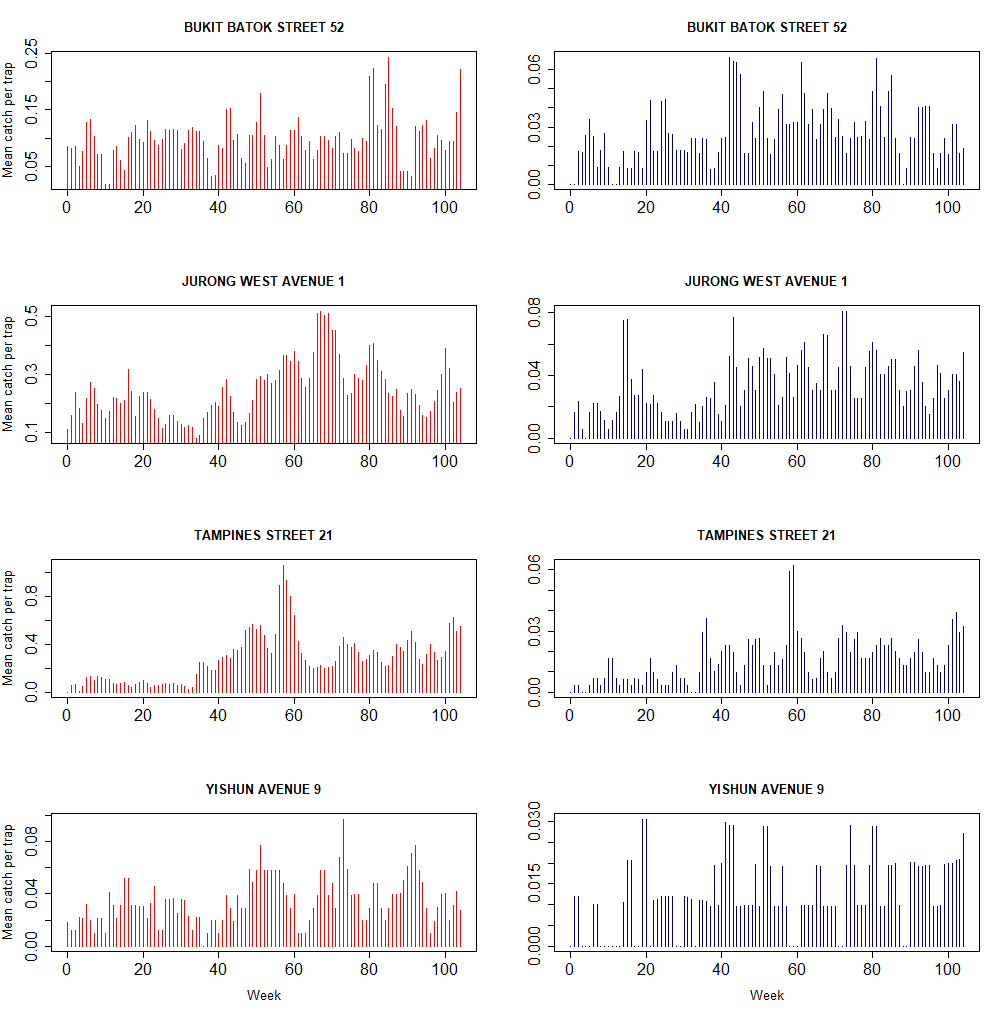


**Figure S2: Visualizations of spatial variables**: (a) distance to the nearest waterway (m), (b) Drain line density (km^-1^), and (c) distance to the nearest water area (m) for each site, as well as (d) the land classification map produced by Richards & Tunçer.

**Figure S3: Visualizations of weather variables.** Each sub-plot shows a weather variable whose weekly value was averaged across all the sites in the study.


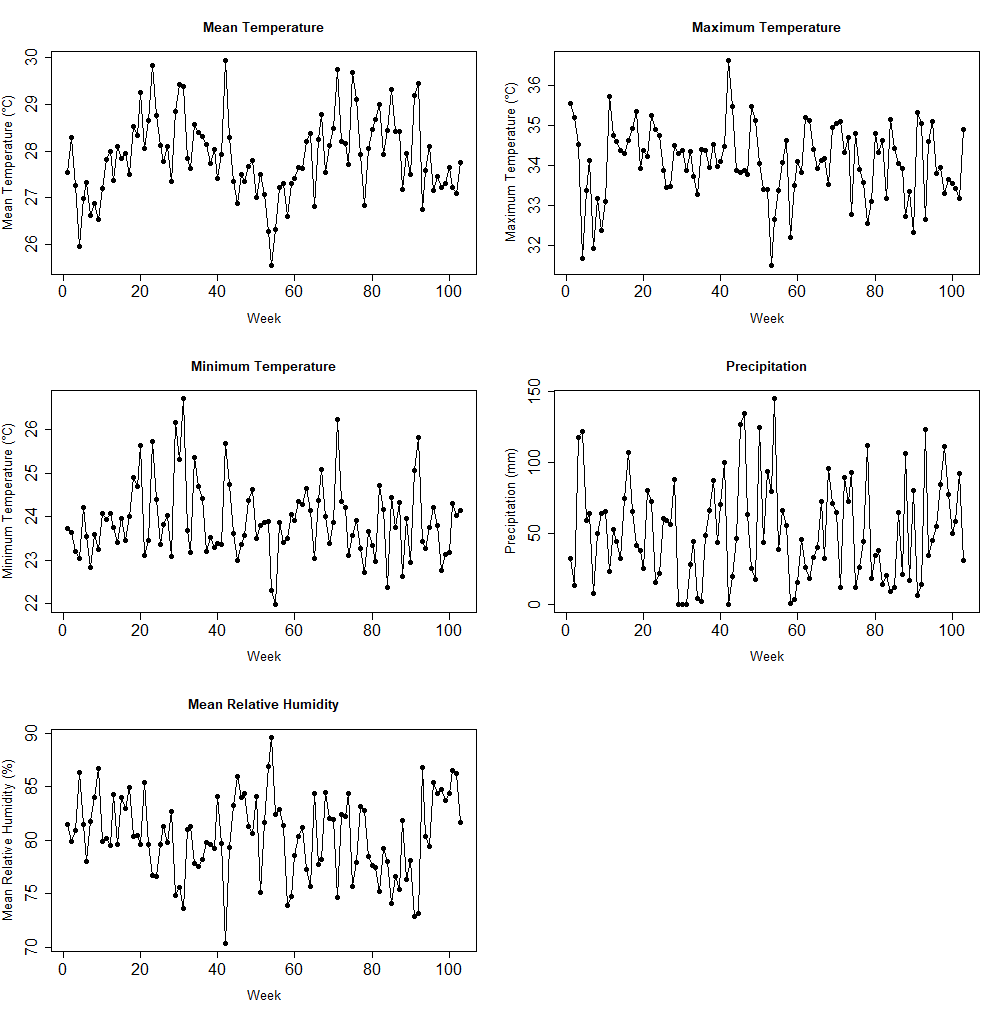

Supplement: Supplementary file 1 — Additional file 1: Figures. [file 13071_2020_4554_MOESM1_ESM.docx]
